# Zoster vaccination is associated with a reduction of zoster in elderly patients with chronic kidney disease

**DOI:** 10.1093/ndt/gfv432

**Published:** 2016-01-13

**Authors:** Sinéad M. Langan, Sara L. Thomas, Liam Smeeth, David J. Margolis, Dorothea Nitsch

**Affiliations:** 1 Faculty of Epidemiology and Population Health, London School of Hygiene and Tropical Medicine, London, UK; 2 Department of Dermatology and Center for Clinical Epidemiology and Biostatistics, University of Pennsylvania, Philadelphia, PA, USA

**Keywords:** chronic kidney disease, herpes zoster vaccine, vaccine effectiveness

## Abstract

**Background:**

Growing epidemiological evidence demonstrates increased zoster risks in people with chronic kidney disease (CKD). Study objectives were to determine zoster vaccine effectiveness in individuals with CKD in pragmatic use.

**Methods:**

A population-based cohort study was undertaken in a 5% random sample of US Medicare from 2007 to 2009 involving 766 330 eligible individuals aged ≥65 years who were (29 785) and were not (736 545) exposed to the zoster vaccine. Incidence rates for zoster in vaccinated and unvaccinated individuals and hazard ratios for zoster comparing vaccinated with unvaccinated were determined for individuals with CKD. Time-updated Cox proportional hazards models were used, adjusting for relevant confounders.

**Results:**

CKD was present in 183 762 (24%) of individuals (15% of vaccinees). Adjusted vaccine effectiveness [95% confidence intervals (CIs)] in individuals with CKD was 0.49 (0.36–0.65). The adjusted vaccine effectiveness in participants with both CKD and diabetes mellitus was 0.46 (95% CI 0.09–0.68). Vaccine effectiveness estimates were similar to those previously reported for the general population [vaccine effectiveness 0.48 (95% CI 0.39–0.56)].

**Conclusions:**

Zoster vaccine is effective against incident zoster in older individuals with CKD. Extra efforts are warranted to increase vaccine uptake in individuals with CKD given the known low uptake in these higher risk individuals.

## INTRODUCTION

Zoster is a major public health problem associated with significant morbidity, including prolonged, severe pain, namely post-herpetic neuralgia (PHN). PHN is usually defined as pain that persists for 3 months or greater following incident zoster [1]. The effective live zoster vaccine was introduced in the USA for immunocompetent individuals aged ≥60 years [2].

Increasing epidemiological evidence supports an increased risk of zoster in individuals with chronic kidney disease (CKD) [3, 4]. For example, we reported that older beneficiaries with CKD were at increased risk of zoster, adjusted hazard ratio 1.16 [95% confidence interval (CI) 1.11–1.21] [5]. There is also evidence to suggest that developing zoster may be detrimental to individuals with CKD [6].

We also showed that older individuals in US Medicare with CKD were less likely to receive the zoster vaccine compared with people without CKD [5]. If the vaccine is similarly effective in people with CKD, the absolute benefits of zoster vaccination will be higher among people with CKD given their higher baseline risk. Therefore, we assessed zoster vaccine effectiveness among people with CKD. Diabetes is the leading cause of end-stage renal disease internationally, and pre-dialysis CKD frequently co-exists with diabetes mellitus; [7, 8] diabetes has also been proposed as a possible risk factor for zoster [9]. Therefore, we additionally assessed vaccine effectiveness in people with CKD and diabetes mellitus.

## MATERIALS AND METHODS

The methods for this study have been described in detail previously [5]; a brief summary is given below.

This study used the 5% random Medicare Standard Analytic Files including Denominator, Inpatient hospital discharge records (MedPAR), Physician/Supplier (Carrier) and Outpatient files from January 2007 to December 2009. The study population were identified as previously described and comprised eligible individuals aged ≥65 years [5]. Individuals start of follow-up was the first date they fulfilled study eligibility criteria (at least 12-months continuous enrolment with eligibility for treatment under Medicare parts A, B and D) with an additional 12 month baseline pre-study observation period added to ensure observation of incident rather than prevalent zoster. The end of follow-up was defined as the earliest of end of eligibility, date of death, development of herpes zoster or the end of the study period. Individuals enrolled in health maintenance organizations were excluded from the study as information on clinical events is not available. Individuals with episodes of herpes zoster in the first year pre-study observation period or those who received the herpes zoster vaccine during the baseline pre-study observation period were excluded from analysis. Exposure to the herpes zoster vaccine was identified based on Current Procedural Terminology (CPT) code 90736 or the National Drug Codes (NDC) for the herpes zoster vaccine. If CPT code 90741 or Healthcare Common Procedural Coding System (HCPCS) code G0377 was present within 7 days of vaccine purchase, that would be considered the date of vaccination, otherwise the date of recording of the NDC code for herpes zoster vaccine was considered to be the administration date. Incident herpes zoster was identified by the presence of a specific International Classification of Diseases, Ninth Revision, Clinical Modification (ICD-9-CM) diagnostic code for herpes zoster, and the use of systemic antiviral therapy, within 7 days either before or after the diagnostic code for herpes zoster [10]. Cases were identified from outpatient, inpatient or healthcare provider (Carrier) files.

CKD, diabetes mellitus and kidney transplants were identified from records by the presence of two or more ICD-9-CM codes for each exposure on different days in the outpatient or carrier files or one or more codes from inpatient records. Chronic dialysis was defined as the presence of at least two dialysis codes separated by 30 days and within 365 days. Covariates were identified as previously described [5].

Incidence rates for zoster by population characteristics were determined by dividing events by person-years of follow-up. Cox regression was used to derive hazard ratios for zoster in vaccinees compared with the unvaccinated, adjusting for age, gender, race, low income, immunosuppression (including immunosuppression related to biologic therapies) and other comorbidities including immune-mediated disorders (systemic lupus erythematosus, inflammatory bowel disease and rheumatoid arthritis) and disorders such as chronic obstructive pulmonary disease, previously reported to increase zoster risk, Age and immunosuppression were included as time-varying covariates. Hazard ratios for zoster in vaccinees compared with the unvaccinated were determined for individuals with CKD, diabetes mellitus and both disorders. To ensure that the findings applied to pre-dialysis CKD, a sensitivity analysis was undertaken excluding individuals with evidence of dialysis or a renal transplant. Vaccine effectiveness (VE) was calculated as VE = 1-hazard ratio of zoster in those vaccinated compared with individuals who did not receive the vaccine.

## RESULTS

The study population comprised 766 330 individuals, of whom 29 785 individuals were vaccinated. CKD was present in 183 762 (24%) of individuals (15% of vaccinees), 300 015 (39.2%) had diabetes mellitus (29% of vaccinees) and 106 026 (14%) had both diseases (7% of vaccinees) (Table 1). Kidney disease stage was available for 42% of the study cohort (Table 1), with the majority of those with identifiable disease stage having stage 3 CKD. A small minority of the population were on renal replacement therapy, with 11 742 individuals on dialysis, of whom 97 (0.3%) were vaccinated and 733 individuals with evidence of a renal transplant, of whom 15 were vaccinated (0.1%).

**Table 1.** Demographics of the study population

Characteristic	Population overall *n* (%)	Individuals vaccinated *n* (%) *n* = 29 785	Individuals not vaccinated *n* (%) *n* = 736 545
Age (years)^a^			
65–69	209 992 (27.4)	8805 (29.6)	201 187 (27.3)
70–74	160 022 (20.9)	7994 (26.8)	152 028 (20.6)
75–79	141 884 (18.5)	6127 (20.6)	135 757 (18.4)
≥80	254 432 (33.2)	6859 (23.0)	247 573 (33.6)
Gender			
Male	247 940 (32.4)	9111 (30.6)	238 829 (32.4)
Female	518 390 (67.7)	20 674 (69.4)	497 716 (67.6)
Race^b^			
White	646 803 (84.4)	27 889 (93.6)	618 914 (84.0)
Black	66 506 (8.7)	387 (1.3)	66 119 (9.0)
Other	52 046 (6.8)	1509 (5.1)	51 512 (7.0)
Low income			
No	570 182 (74.4)	27 405 (92.0)	542 777 (73.7)
Yes	196 148 (25.6)	2380 (8.0)	193 768 (26.3)
CKD			
No	582 568 (76.0)	25 261 (84.8)	557 307 (75.7)
Yes	183 762 (24.0)	4524 (15.2)	179 238 (24.3)
Stage unavailable	107 101 (58.3)	2661 (8.9)	104 440 (14.2)
CKD Stage 1	4702 (0.6)	136 (0.5)	4566 (0.6)
CKD Stage 2	8348 (1.1)	274 (0.9)	8704 (1.1)
CKD Stage 3	37 142 (4.9)	1123 (3.8)	36 019 (4.9)
CKD Stage 4	10 039 (1.3)	166 (0.6)	9.873 (1.3)
CKD Stage 5	16 430 (2.1)	164 (0.1)	16 266 (2.2)
CKD Stage 5—on renal replacement therapy			
On dialysis	11 742 (1.5)	97 (0.3)	11 645 (1.6)
Renal transplantation	773 (0.1)	15 (0.1)	758 (0.1)
Diabetes mellitus			
No	466 315 (60.9)	21 029 (70.6)	445 286 (60.5)
Yes	300 015 (39.2)	8756 (29.4)	291 259 (39.5)
CKD and diabetes mellitus			
No	660 304 (86.2)	27 569 (92.6)	632 735 (85.9)
Yes	106 026 (13.8)	2216 (7.4)	103 810 (14.1)

^a^For determination of numbers, age at vaccination used if vaccinated; otherwise baseline age.

^b^Missing race information for 975 people (0.1%).

Zoster incidence rates were higher in unvaccinated individuals with CKD (11.4 per 1000 person-years, 95% CI 11.0–11.8) compared with the unvaccinated population overall (10.0 per 1000 person years, 95% CI 9.8–10.2). Zoster incidence was increased to a similar extent in those unvaccinated individuals with both CKD and diabetes mellitus (11.0 per 1000 person years, 95% CI 10.5–11.5; Table 2), compared with the unvaccinated population.

**Table 2.** Vaccine effectiveness in people with chronic kidney disease and diabetes mellitus

Vaccination status	Events	Person-years (py) (1000)	Incidence rate per 1000 py (95% CI)	Crude hazard ratio (95% CI)	Adj. hazard ratio^a^ (95% CI)
Unvaccinated overall	12 958	1291.8	10.0 (9.8–10.2)	1.0	1.0
Vaccinated overall	154	28.3	5.4 (4.6–6.4)	0.55 (0.47–0.64)	0.52 (0.44–0.61)
Unvaccinated CKD	3438	302.0	11.4 (11.0–11.8)	1.0	1.0
Vaccinated CKD	28	4.4	6.4 (4.4–9.2)	0.56 (0.39–0.81)	0.51 (0.35–0.74)
Unvaccinated diabetes mellitus	5181	509.2	10.2 (9.9–10.4)	1.0	1.0
Vaccinated diabetes mellitus	46	8.4	5.4 (4.1–7.3)	0.54 (0.40–0.72)	0.50 (0.38–0.67)
Unvaccinated CKD and diabetes mellitus	1926	174.9	11.0 (10.5–11.5)	1.0	1.0
Vaccinated CKD and diabetes mellitus	14	2.2	6.5 (3.8–10.9)	0.59 (0.35–1.00)	0.54 (0.32–0.91)

^a^Adjusted for age, gender, race, low income, immunosuppression, other comorbidities including immune-mediated disorders (systemic lupus erythematosus, inflammatory bowel disease and rheumatoid arthritis) and chronic obstructive pulmonary disease, with age and immunosuppression as time-varying covariates.

Amongst those with CKD, the zoster incidence rate in those not vaccinated was nearly double that in vaccinees (11.4 per 1000 person-years, 95% CI 11.0–11.8 in vaccinees with CKD, compared with 6.4 per 1000 person years, 95% CI 4.4–9.2 in those with CKD who were not vaccinated; Table 2). Adjusted VE in individuals with CKD was 0.49 (95% CI 0.36–0.65). Among individuals with both CKD and diabetes mellitus, incidence rates in those not vaccinated were 11.0 (95% CI 10.5–11.5) and 6.5 per 1000 person-years (95% CI 3.8–10.9) in vaccinees, giving an adjusted VE of 0.46 (95% CI 0.09–0.68) (Table 2). A sensitivity analysis excluding beneficiaries with evidence of either renal transplants or dialysis did not alter study findings (data not shown).

## DISCUSSION

Zoster incidence rate was higher in those with CKD and in those with CKD and diabetes mellitus compared with individuals without these conditions. Zoster VE in beneficiaries with CKD and with both CKD and diabetes mellitus was similar to VE in the overall study population [vaccine effectiveness 0.48 (95% CI 0.39–0.56)] [5].

Medicare beneficiaries are reasonably representative of the older US population; 98% of Americans aged ≥65 years were enrolled in Medicare in 2009 [11]. Our study is large, giving power to assess VE in individuals with CKD. Despite having adequate power to determine VE in the CKD cohort, our study size was insufficient to enable study of VE against PHN. Additionally, there were too few events amongst individuals on dialysis or with transplants to enable study of VE in these subgroups. VE was determined after adjusting for a wide range of confounders. Medicare is an administrative data source, hence there could be misclassification of exposures and outcomes which may have led to a bias towards the null.

Information on CKD stage was only available for 42% of individuals with CKD, hence it is not possible to comment on the VE of the zoster vaccine by CKD stage. Additionally, the study period was relatively short (maximum 2 years), due to data costs and availability issues, hence we could not determine long-term vaccine effectiveness. Although the vaccine was licensed in 2006 and recommended by the Advisory Committee on Immunization Practices later that year, publication of the recommendation to vaccinate was delayed, which may have contributed to the low uptake of this vaccine. Uptake of the zoster vaccine remains low, but has gradually increased over time [12].

We have previously found that individuals with CKD were less likely to have received the zoster vaccine than people without CKD [5]. Previous studies have also reported increased incidence of zoster in adults with CKD and in those being treated with dialysis compared with those without CKD [3, 4, 13]. A recent retrospective cohort study from Taiwan reported a 27% increased zoster incidence in those with CKD compared with those without CKD and higher risks in those receiving renal replacement therapy [4]. To our knowledge, no previous study has assessed the VE of zoster vaccine in individuals with CKD. Good clinical and serological responses have been reported for the varicella vaccine in CKD in paediatric populations; however, the dose of the latter vaccine is 14 times lower than the zoster vaccine with distinct recommendations for use [2, 14, 15].

CKD is associated with immunosuppression with increasing immune defects, including reduced naïve and memory T cells in association with declining renal function. In those with end-stage renal disease, infection is a cause of significant morbidity and mortality, and is second only to cardiovascular disease as a cause of death [16]. Defective cellular and humoral immunity in CKD and end-stage renal disease are not only contributing factors to the predisposition to develop infections, but may also lead to reduced VE [17]. Previous studies have reported reduced seroconversion rates and lower and more quickly declining antibody titres in patients with CKD, leading to the requirement to use booster doses and suboptimal vaccine responses; booster doses are not recommended for the zoster vaccine [17].

We have shown that, despite reduced vaccine uptake in people with CKD and in those with CKD coexisting with diabetes mellitus, and despite concerns about reduced effectiveness in CKD, the zoster vaccine is effective in these beneficiaries with similar levels of effectiveness to the general older US population. Extra efforts are warranted to increase vaccine uptake in people with CKD in the USA and in the UK according to guidelines. The greater absolute benefits from vaccinating people with CKD should inform cost-effectiveness analyses and influence vaccination policies. This novel timely study is of particular relevance in the early stages of introducing a large scale vaccination programme.

## CONFLICT OF INTEREST STATEMENT

L.S. has undertaken consultancy for GlaxoSmithKline. D.J.M. is on separate data safety monitoring boards for Abbott and Astellas; the remaining authors state no conflicts of interest. The results presented in this paper have not been published previously in whole or part, except in abstract format.
